# TEMPO-Oxidized Nanocellulose Films Modified by Tea Saponin Derived from *Camellia oleifera*: Physicochemical, Mechanical, and Antibacterial Properties

**DOI:** 10.3390/polym16071016

**Published:** 2024-04-08

**Authors:** Nan Jiang, Yudi Hu, Yuhang Cheng

**Affiliations:** School of Packaging and Materials Engineering, Hunan University of Technology, Zhuzhou 412007, China; judy_huyudi@163.com (Y.H.); chengyuhang3319@163.com (Y.C.)

**Keywords:** TEMPO oxidation, nanocellulose, tea saponins, antibacterial property, oxygen barrier

## Abstract

Nanocellulose materials have been widely used in biomedicine, food packaging, aerospace, composite material, and other fields. In this work, cellulose obtained from Camellia shells through alkali boiling and subbleaching was micro-dissolved and regenerated using the DMAc (*N*,*N*-Dimethylacetamide)/LiCl system, and TOCNs (TEMPO-oxidized cellulose nanofibers) with different degrees of oxidation. The membrane was prepared by filtration of polytetrafluoroethylene (pore size 0.1 μm), and the oxidized nanocellulose film was obtained after drying, Then, the crystallinity, mechanical properties and oxygen barrier properties of the TOCN film were investigated. Furthermore, based on TS (tea saponin) from *Camellia oleifera* seed cake and TOCNs, TS-TOCN film was prepared by the heterogeneous reaction. The TS-TOCN film not only shows excellent oxygen barrier properties (the oxygen permeability is 2.88 cc·m^−2^·d^−1^) but also has good antibacterial effects on both Gram-negative and Gram-positive bacteria. The antibacterial property is comparable to ZnO-TOCN with the same antibacterial content prepared by the in-situ deposition method. Antioxidant activity tests in vitro showed that TS-TOCN had a significant scavenging effect on DPPH (2,2-Diphenyl-1-picrylhydrazyl) radicals. This design strategy makes it possible for inexpensive and abundant *Camellia oleifera* remainders to be widely used in the field of biobased materials.

## 1. Introduction

The shortage of non-renewable resources and environmental pollution are two kinds of crises caused by the rapid development of industry and the over-consumption of petroleum-based polymers in our daily lives. The development of biobased degradable materials could not only fundamentally solve environmental pollution but also substantially reduce oil consumption and relieve pressure on petrochemical resources [1,2]. Biomass materials have potential application value because they are the most plentiful renewable resource on earth [3]. Biomass can be converted into chemicals, fuels, and carbon materials via a variety of techniques, including biochemical [4], thermo-chemical [5], and physio-chemical processes [6]. 

Cellulose is a kind of natural biological material and is the most abundant renewable biopolymer on the planet. The nanocellulose prepared from native cellulose has been widely used as biomedical carriers and wound dressings [7,8], flexible wearable materials [9,10], and packaging materials [11,12] for premium products due to their low cost, biodegradability [13], great biocompatibility [14], and desirable physical properties [15]. Crude fibers obtained from plants through alkali boiling and subbleaching were subjected to pretreatment with DMAC/LiCl for subsequent oxidation [16]. The DMAc/LiCl mixture represents a well-established cellulose dissolution system. The cellulose solution can keep a state of high chemical stability in the DMAc/LiCl system, and the dissolved cellulose can be easily regenerated in a water bath [17]. The cellulose prepared by oxidation can be easily made into nanocellulose by mechanical treatment. The TEMPO-mediated oxidation system is an effective cellulose selective oxidation system that has attracted enormous investigation due to its unique advantages, such as recyclability, high selectivity, and efficient nano-exfoliation [18,19,20]. The oxidized cellulose prepared by the TEMPO/NaBr/NaClO system has a high aspect ratio, and TEMPO-oxidant cellulose functional materials have a high oxygen barrier and mechanical properties. Akira Isogai [21] prepared completely individual TEMPO-oxidant cellulose nanofibers that had homogeneous widths of 3 nm and long lengths of 500 nm, which implies that materials based on TEMPO-oxidant cellulose have fantastic comprehensive properties. In addition, TOCN film has excellent oxygen barrier properties and has potential as a substrate for a high barrier material, such as the oxygen permeability of PLA film, which can be reduced to 0.13% after coating with oxidized nanocellulose [22]. Moreover, TOCN films also have excellent mechanical properties [23]; the tensile strength and strain of the production at break are 233 MPa and 4.5%, respectively. Therefore, TOCNs show great potential for applications in the food and pharmaceutical fields.

TSs are a group of glycoside compounds extracted from plants of the tea family, consisting of glycosides and aglycones associated with sterols or triterpenes. It is a class of compounds [24] with pharmacological activities such as antioxidant [25,26,27], antibacterial [28,29], anticancer [30,31], and hypocholesterolemia [32] properties. The antibacterial activity of tea saponins has attracted much attention. Avato P. et al. [33] found that the antimicrobial activity of saponins from M. arabica was especially high against Gram-positive bacteria. TS can inhibit the growth of bacteria via regulating the transcription of genes [34]. In addition, the DPPH radical-scavenging activity of TS at 6 mg/mL is 57.33% [35], indicating that TS has the potential to be a kind of natural antioxidant with excellent properties. However, there are few studies on the preparation of functional materials using TS.

**Figure 1:**
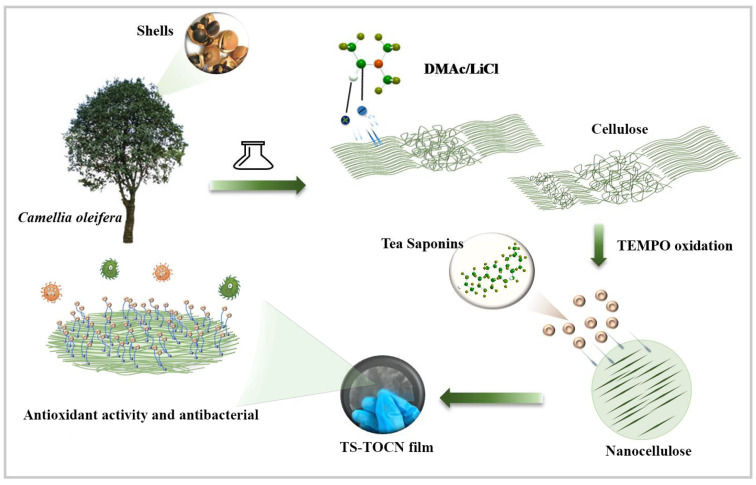
Schematic presentation of TS-TOCN synthesis and property.

The development and preparation of purely natural biobased antimicrobial cellulose materials remain a major challenge. The effect of DMAc/LiCl on the properties of oxidized nanocellulose films was studied in this work, and on this basis, a new antibacterial film was prepared from *Camellia oleifera* remainders (Figure 1). The carboxyl content of TOCNs was increased by micro-soluble regeneration, realizing the high grafting rate of TS on TOCN film. The antibacterial and antioxidant properties of TS-TOCN film were verified, which will provide a scientific basis for the application of TS-TOCN films in the food and medical fields.

## 2. Materials and Methods

### 2.1. Materials

Cellulose was obtained in the laboratory from fresh *Camellia oleifera* shells; tea saponins were extracted by the laboratory of Central South University of Forestry and Technology from Camellia seed cake [36], and the total aglycone extracts were used in this work. Sodium bicarbonate, sodium hypochlorite, and lithium chloride were purchased from Anhui Zesheng Technology Co., Ltd., Anhui, China; DMAc(*N*,*N*-dimethylacetamide) and DMAP(4-dimethylaminopyridine) were purchased from Shanghai Titan Technology Co., Ltd., Shanghai, China; sodium hydroxide, 2,2,6,6-tetramethylpiperidine 1-oxygen radical (TEMPO), sodium bromide, zinc acetate, and absolute ethanol were purchased from Shanghai Aladdin Biochemical Technology Co., Ltd., Shanghai, China; *Escherichia coli* (*E. coli*) and *Staphylococcus aureus* (*S. aureus*) were purchased from Shanghai Luwei Technology Co., Ltd., Shanghai, China; and silver nitrate was purchased form Sinopharm Chemical Reagent Co., Ltd., Shanghai, China.

### 2.2. Pretreatment Method

#### 2.2.1. Preparation of Cellulose

The primary chemical components of *Camellia oleifera* fruit shells are cellulose, hemicellulose, and lignin, the same major constituents as wood [37]. Cellulose was obtained by alkali boiling and subbleaching from fresh *Camellia oleifera* shells by the following steps: The shells were cleaned with deionized water to remove mud and impurities, followed by placement in a blast drying oven at 60 °C for 4 h. Subsequently, the dried shells were ground and sieved using a 60-mesh sample sieve. The sifted shell powder (10 g) was added to a 200 mL solution of 2% NaOH. After reacting at 80 °C for 4 h, the solution was removed through vacuum filtration and subjected to repeated washing. The yield of alkaline-treated fruit shell powder was 52%. Subsequently, the powder (10 g) was transferred to a 200 mL solution containing 5% sodium hypochlorite, and the pH of the mixed solution was adjusted to 4.5 using acetic acid. The mixture underwent a bleaching reaction at 80 °C for 4 h followed by cooling and repeated filtration and washing once the reaction was complete. Then, delignified and dehemicellulosed cellulose were obtained with a yield of 39%.

By employing the DMAc/LiCl system as a pretreatment method for cellulose, it becomes feasible to disrupt the crystalline region of cellulose and augment the amorphous area, thereby effectively enhancing the efficiency of cellulose oxidation. Based on Roder et al.’s research findings, we opted for four distinct mass concentrations of LiCl to conduct our experiments. The steps were as follows: 200 mL of DMAc, LiCl (3 wt%, 5 wt%, 7 wt%, 9 wt%), and 3 g of cellulose were added into four 500 mL beakers. The mixture system was heated to 100 °C and stirred for 1 h, and after cooling at room temperature, it continued to be stirred for 12 h. Then, 10 L of ultrapure water was added to the cellulose suspension solution dropwise and stirred vigorously. After being filtered and freeze-dried for 3 days, yielding 83% DMAc/LiCl-treated cellulose, the LiCl concentrations of 3 wt%, 5 wt%, 7 wt%, and 9 wt% were obtained and named C-3, C-5, C-7, and C-9 (C-*x*), respectively, while the untreated fibers were named C-0.

The cellulose and hemicellulose content in the samples was determined according to the industry standard of United States TAPPIT257om-09. The determination of lignin content, ash content, and extract content referred to the standards of United States TAPPIT222 om-11, TAPPIT211 om-12, and TAPPIT207 cm-08, respectively [38]. 

#### 2.2.2. Preparation of TOCNs

The prepared sample C-*x* was suspended in water (300 mL) containing TEMPO (0.3 g) and sodium bromide (0.36 g). Then, 0.1 mol·L^−1^ HCl was added to adjust the pH of the 12% (*v*/*v*) NaClO solution to 10. The NaClO solution (ratio of 1.4 mmol NaClO to 1 g of cellulose) was added to the mixture by drops and stirred at room temperature. The pH of the mixed solution was maintained at 10 by continuous addition of the NaOH solution (0.5 mol·L^−1^). When no more decrease in pH was observed, the reaction was finished, and the pH was adjusted to 7 by adding 0.1 mol·L^−1^ of HCl. The TEMPO-oxidized cellulose was thoroughly washed with water by filtration. The purified cellulose was dispersed in water at a concentration of 1.08 wt% and then disintegrated by successively passing through a high-pressure homogenizer for three cycles at a pressure of 80 MPa and 25 °C. A series of TOCN aqueous dispersions were ultimately obtained and were denoted as “TOCN-*x*”, in which the number for “*x*” refers to the concentration of the DMAc/LiCl system.

#### 2.2.3. Preparation of TOCN Film

The TOCN-*x*/water dispersion was converted to a film with about 1 mm thickness on a surface hydrophilized polytetrafluoroethylene (PTFE) membrane with 0.1 µm pore size by suction filtration. The TOCN-*x* film was dried in a ventilated oven at 50 °C overnight without forced airflow.

#### 2.2.4. Preparation of TS-TOCN Film

The content of the carboxyl group in the composite film was determined according to the mass of TOCN-*x* in the film preparation process to determine the maximum grafting content of TS. The TOCN-*x* film with the best comprehensive properties among the prepared TOCN films was selected for TS modification.

The selected TOCN-*x* film was immersed in the aqueous solution of TS (0.4 wt% 25 mL), 0.1 g of DMAP was added as catalyst, and the reaction was carried out at room temperature for 8 h. Repeat washing with deionized water was conducted to remove the DMAP catalyst. The TS-TOCN film was prepared. In addition, the stable and efficient antibacterial properties of composite films containing Nano-ZnO and Nano-Ag have been extensively demonstrated in numerous studies [39,40]. ZnO-TOCN and Ag-TOCN films were prepared via in-situ deposition and used as the reference against which the antibacterial properties of the TS-TOCN films were analyzed.

### 2.3. Performance Testing and Characterization

#### 2.3.1. Sample Morphology Observation

Morphology of the prepared samples was observed using a Field Emission Scanning Electron Microscope (FESEM) (Carl Zeiss AG, Oberkochen, Germany, FE-SEM ZEISS Sigma 300), and the samples were sputter coated with a layer of gold using the sputtering technique before analysis. The analysis was performed at an accelerating voltage of 5 kV. Similarly, the TOCN film cross section was prepared via the liquid nitrogen refrigeration method.

#### 2.3.2. X-ray Diffraction (XRD)

The XRD spectra for freeze-dried nanocellulose was measured using an X-ray polycrystalline diffractometer (Rigaku, Tokyo, Japan, Ultima IV) with a Cu Kα X-ray source (λ = 0.1548 nm) at 40 kV and 15 mA. The crystallinity index (*CrI*) was calculated by the Segal method in Equation (1):

(1) $$CrI=\frac{I_{002}-I_{amorphous}}{I_{002}}\times 100\%$$

where *I*_002_ is the intensity of the main peak (002) and *I_amorphous_* is the intensity of the amorphous portion at 2θ = 18.0°.

#### 2.3.3. Determination of Carboxyl in TOCN-*x*

The content of the carboxyl group in the oxidized cellulose was determined by the TAPPI T237 cm-98 (2006) standard. An appropriate amount of oxidized cellulose was taken, treated with a 0.1 mol/L hydrochloric acid solution for 120 min, and then washed with deionized water until the filtrate was neutral. The wet pulp cake was accurately weighed (accurate to 0.1 g) and placed in a conical bottle. Then, 50 mL of a pre-prepared NaHCO_3_-NaCl solution was quickly added into the conical flask, and the reaction was carried out for 60 min before filtration. Taking 25 mL of filtrate, 1–2 drops of methyl red indicator was added into it, then 0.01 mol/L hydrochloric acid solution was used for titration. The carboxyl group content was calculated by Formula (2):

(2) $$\mathrm{carboxyl}\;\mathrm{group}\;\mathrm{content}\;(\mathrm{meq}/100\;g)=\{B-[A+(A\;\times \;\frac{C}{50})]\}\;\times \;N\;\times \;\frac{200}{W}$$

where *A* is the amount of hydrochloric acid solution when titrating 25 mL of filtrate, mL; *B* is the amount of hydrochloric acid solution when titrating the NaHCO_3_-NaCl solution, mL; *C* is the mass of water in the wet cake, g; *N* is the actual concentration of the hydrochloric acid solution used for titration, mol/L; and *W* is the absolute dry mass of the sample, g.

#### 2.3.4. Fourier-Transform Infrared Spectroscopy (FT-IR)

Infrared spectra of the TOCN-*x* and TS-TOCN were measured using Fourier-transform infrared spectroscopy (Bruker, karlsruhe, Germany, FT-IR TENSOR II spectrometer) with a resolution of 4 cm^−1^. The dried samples (powder form) were mixed well with KBr powder and pressed into an ultra-thin pellet with a ratio of 1:10 (*w*/*w*). The spectra were collected at ambient conditions in the transmittance mode over the regions of 4000–400 cm^−1^.

#### 2.3.5. Tensile Test

Tensile strengths and Young’s moduli of films 5 mm in wide and at least 40 mm in length were measured at 1.0 mm min^−1^ and 20 mm span length using a Universal Testing Machine (Shenzhen Wance, Shenzhen, China, ETM-104B) equipped with a 500 N load cell. At least three specimens were tested for each sample and averaged.

#### 2.3.6. Antibacterial Activity Testing

*Escherichia coli* (*E. coli*) and *Staphylococcus aureus* (*S. aureus*) were cultured in Tryptic Soy Broth (30 g/L) at 35 °C for 24 h and used to test the antibacterial activity of the TS-TOCN films in this study. The prepared films of TS-TOCN, ZnO-TOCN, and Ag-TOCN were cut into 2 × 2 cm samples. Each sample and 3 mL of the inoculum were added to 10 mL tubes and incubated for 24 h at 35 °C in a shaking water bath. The inhibitive property of the films was calculated using the dilution plate method. The films were washed repeatedly with 75% *v*/*v* ethanol after co-culture, irradiated under a UV lamp for 30 min, and co-cultured with the bacterial solution again. This process was repeated 3 times.

#### 2.3.7. Oxygen Permeability Analysis

The oxygen permeability determinations of TOCN-*x* and TS-TOCN films were averaged three times using an oxygen transmittance tester (Modern Controls Inc., AMETEK MOCON, Minneapolis, MN, USA, MOCON OX-TRAN Model 2/10) instrument under dry conditions according to the standard method (ASTM D3985) [41,42].

#### 2.3.8. Antioxidant Property Analysis

DPPH was mixed with anhydrous methanol to prepare a 0.01 mg/mL DPPH methanol solution. TOCN-5 and TS-TOCN were cut into small pieces and mixed with 5 mL of a DPPH methanol solution. The mixture was kept at room temperature in the dark for 60 min. The absorbance was measured at 517 nm using an ultraviolet visible spectrometer (Varian, Palo Alto, CA, USA, Cary 4000). The DPPH methanol was used as control. The DPPH radical scavenging activity was calculated by using Equation (3):

(3) $$(RSA,\;\%)=(\frac{A_{control}-A_{sample}}{A_{control}})\times 100\%$$

where *A_sample_* is the absorbance of the sample and *A_control_* is the absorbance of the control.

## 3. Results and Discussion

### 3.1. Chemical Composition of Celluloses

The chemical composition of *Camellia oleifera* fruit shells and its products during treatment are shown in Table 1. The untreated Camellia shell contained 29.1% lignin, 28.6% hemicellulose, and 19.2% cellulose, with a holocellulose content of 47.9%, indicating its potential as a raw material for nanocellulose preparation. After undergoing alkali treatment and sodium hypochlorite bleaching, the non-fiber components, such as lignin and hemicellulose, were significantly removed from the *Camellia oleifera* fruit shell, resulting in an increase in the *Camellia oleifera* fruit shell cellulose content to 84.3% by mass. It is evident that the cellulose content increases significantly following treatment with various concentrations of DMAC/LiCl. The purity of cellulose treated with DMAc/LiCl (LiCl 5 wt%) reached an impressive level of 96.7%.

### 3.2. Surface Morphology Analysis

**Figure 2:**
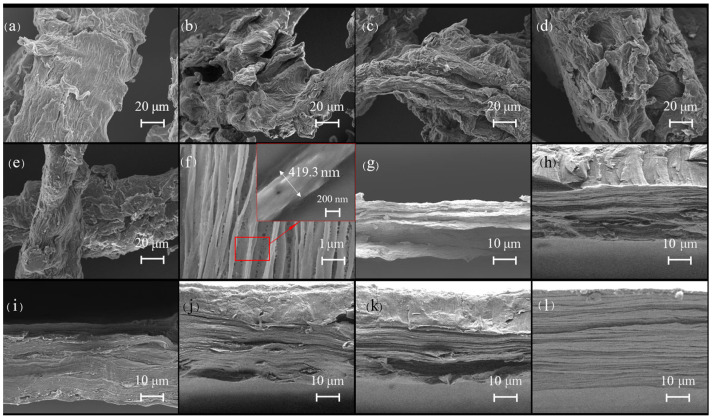
SEM scan of surface topography (**a**–**e**): (**a**) untreated cellulose C-0; (**b**) DMAc/LiCl (LiCl 3 wt%) treated cellulose C-3; (**c**) DMAc/LiCl (LiCl 5 wt%) treated cellulose C-5; (**d**) DMAc/LiCl (LiCl 7 wt%) treated cellulose C-7; (**e**) DMAc/LiCl (LiCl 9 wt%) treated cellulose C-9; and (**f**) average diameter of TOCN and SEM scan of cross section of TOCN film (**g**–**l**). (**g**) TOCN-0; (**h**) TOCNs-3; (**i**) TOCNs-5; (**j**) TOCNs-7; (**k**) TOCNs-9; and (**l**) TS-TOCN film.

The SEM images directly reflected the morphological changes the fruit shell fibers before and after treatment with the DMAc/LiCl system (Figure 2a–e). Electron microscope scanning results showed that the surface of the DMAc/LiCl-treated cellulose (Figure 2b–e) was rougher and shorter in length compared to native cellulose (Figure 2a), which provides a greater possibility for subsequent oxidation reactions, and the higher the concentration of LiCl in the dissolved system, the more obvious the treatment effect on cellulose. The surface of the treated cellulose was rougher due to the effective removal of lignans [37] and the swelling of cellulose by the DMAc/LiCl system [43]. The mechanism of the DMAc/LiCl system on cellulose may involve an initial complexation reaction between Li^+^ and the carbonyl group as well as the nitrogen atom in DMAc. This is followed by the subsequent release of free Cl^−^, which then forms an intermediate complex with the hydroxyl group in cellulose, effectively diminishing the hydrogen bond between cellulose molecules and resulting in the crystalline region of the cellulose being destroyed [44]. However, the TOCNs displayed an ultra-fine fiber with an average diameter of 419.3 nm (Figure 2f), indicating that nanoscale cellulose fibers were successfully prepared in this work.

The cross-sectional morphology of the TOCN-*x* and TS-TOCN films that fractured after freezing with liquid nitrogen were determined by FESEM, as displayed in Figure 2g–k. TOCN film presents a layered structure with a tight internal structure. The lamellar structure inside the TOCN-3 and TOCN-5 films exhibited high densities and orderly layering arrangement (Figure 2h,i). Nevertheless, the lamellar structure of TOCN-7 and TOCN-9 films showed a significant decrease and micropores appeared (Figure 2j,k). This could be due to the reduced cellulose crystallinity achieved after treatment with high-concentration DMAc/LiCl, excessive oxidation efficiency during the TEMPO oxidation process, and the elevated carboxyl content of TOCNs [45], which led to severe material degradation. Consequently, the tensile elongation at break of TOCN-7 and TOCN-9 films demonstrated a declining trend in subsequent film performance testing. TS-TOCN film (Figure 2l) has a similar internal structure as the film before grafting (Figure 2i), which was due to the TEMPO oxidation method that can effectively weaken the cohesion between fiber microfilaments and promote the water absorption and swelling of fibers. TS molecules can utilize the water absorption and swelling phenomenon of cellulose to enter the TOCN films and undergo an esterification reaction with carboxyl groups on the surface and inside of TOCN films to achieve modification of the thin films.

### 3.3. XRD Analysis

**Figure 3:**
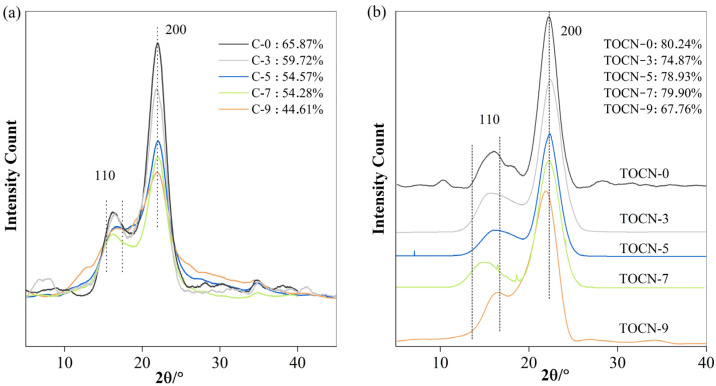
(**a**) XRD spectra of C-*x*; (**b**) XRD spectra of TOCN-*x*.

The X-ray diffraction patterns of C-*x* and TOCN-*x* are illustrated in Figure 3a,b. In these crystallographic patterns, a distinct strong peak at 2θ = 22.4° corresponding to the (200) lattice plane was observed along with two overlapping peaks of (1′1′0) and (110) located at 14° and 17°, respectively, which are the characteristic of cellulose I allomorph. The diffraction peak intensity of all treated celluloses was the only aspect that exhibited changes, suggesting that the DMAc/LiCl system has the ability to preserve the inherent crystal structure of cellulose while simultaneously destroying its crystalline region. The crystallinity of cellulose C-0, the DMAC/LiCl-untreated and unoxidized *Camellia oleifera* shell cellulose extracted from Camellia fruit shells, was determined to be approximately 65.87%. However, as the concentration of LiCl increased to 3 wt%, 5 wt%, 7 wt%, and 9 wt%, the cellulose crystallinity gradually decreased to 59.72%, 54.57%, 54.28%, and 44.61%, respectively (Figure 3a). The decrease can be attributed to the formation of intermediate complexes between the DMAc/LiCl solution and cellulose, which broke both the intermolecular hydrogen bonds of the cellulose and the crystalline region of the cellulose [46]. The crystallinity of all TOCN-*x* samples increased to 80.24%, 74.87%, 78.93%, 79.90%, and 67.76%, respectively (Figure 3b). The observed phenomenon can be further explained by considering that the oxidation reaction preferentially occurs at amorphous domains within fibers, where a higher number of primary hydroxyl groups are oxidized, resulting in the dissolution of water-soluble glucuronic acid. This leads to an increased exposure of crystalline domains within fibers. A similar phenomenon appeared in Hassan’s study [47]. The fundamental reason for the crystallinity decrease in C-9 and TOCN-9 was the extensive depolymerization occurring during pretreatment with the 9% DMAc/LiCl concentration [17].

### 3.4. Effect of Carboxyl Group Content on Cellulose

The –COOH content of TOCN-*x* exhibited a significant increase following pretreatment with DMAc/LiCl (Table 2). Only the primary hydroxyl group in both the crystalline surface and amorphous region of cellulose underwent reaction during oxidation by the TEMPO oxidation system. The presence of hydrogen bonds hindered the reaction, resulting in a gradual decrease in the carboxyl group content [48]. DMAc/LiCl not only facilitates the removal of non-cellulose impurities through the dissolution reaction (Table 1) but also destroys the crystalline region of cellulose, thereby exposing more reactive carboxyl groups. The carboxyl content of the oxidized cellulose prepared via pretreated cellulose was about 1.05–1.89 mmol/g. Han et al. observed that this system effectively increased the amorphous region and reduced the crystallinity while processing cellulose using the DMAc/LiCl system to prepare regenerated films [16].

### 3.5. FT-IR Analysis

**Figure 4:**
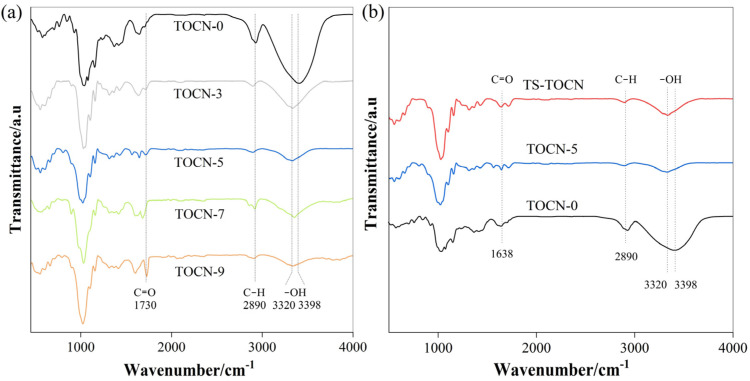
(**a**) FT−IR spectra of TOCN-*x* film and (**b**) FT-IR spectra of TS-TOCN film and the controls.

Figure 4a,b illustrates the FT-IR spectra obtained from both the TOCN-*x* and TS-TOCN samples. The broad peaks observed at 3320–3398 cm^−1^ can be ascribed to the stretching vibration of the hydroxyl group in cellulose, while the peaks at 2897 cm^−1^ are due to the stretching vibration of the C–H group in cellulose. It is evident that even after treatment with DMAc/LiCl, the main characteristic peaks of cellulose remained intact, indicating minimal structural changes. This observation was further supported by XRD analysis of TOCN-5, which showed no obvious alteration in crystallinity compared to the TOCN-7 cellulose. Consequently, there was no substantial change in carboxyl content between TOCN-5 and TOCN-7 when compared to TOCN-0 alone. Relative to TOCN-0, an enhanced band at 1730 cm^−1^ corresponding to the C=O stretching vibration of the carboxyl group was observed for TOCN-3/5/7/9 following treatment with the DMAc/LiCl system, which indicates that the hydrogen bond between free hydroxyl groups was effectively destroyed, and the degree of oxidation was increased.

Furthermore, a decrease in intensity and shift towards the wavenumber position was observed for the O–H stretching vibration peak in the TOCN samples, indicating strengthened molecular hydrogen bonding within functional groups. In terms of TS-modified TOCN, a higher peak at 1730 cm^−1^ indicated successful grafting of TS onto the nanocellulose macromolecules. Additionally, an increase in the peak at 3350 cm^−1^ was attributed to the abundant hydroxyl groups present in TS forming strong hydrogen bonds with the cellulose.

### 3.6. Mechanical Property Analysis

**Figure 5:**
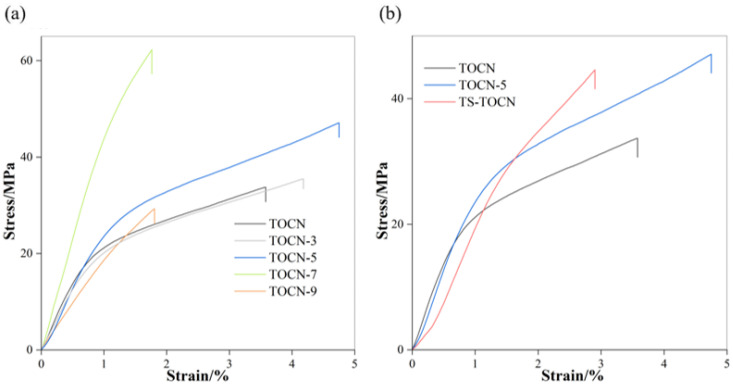
(**a**) Stress–strain curves of TOCN-*x* film; (**b**) stress–strain curves of TS–TOCN film.

The stress–strain curves of the TOCN films (Figure 5a) are typical of a brittle fracture. The brittleness of TOCN films can be attributed to the large aspect ratio, high crystallinity, good dispersion, and the formation of a rigid nanofiber network through hydrogen bonding between adjacent nanofibers [49]. TOCN-7 and TOCN-9 exhibited relatively low strain (strain ≤ 2%) without significant yielding. On the other hand, TOCN-0, TOCN-3, and TOCN-5 initially yielded at a strain of approximately 1% followed by a linear and significantly strain-hardening zone until reaching a strain to failure of 3.58%, 4.18%, and 4.75%, respectively. This behavior is believed to be associated with the onset of interfibril debonding and nanofibril slippage [22]. The higher carboxyl group content in TOCNs leads to easier separation of cellulose, increased fibrillation levels [50], and a denser nanofiber network, consequently enhancing the tensile strength of the membrane with the increasing oxidation level. C-7 experienced partial dissolution during pretreatment, which resulted in a lower aspect ratio but significantly increased tensile strength while reducing the elongation at break [51]. However, this work observed that C-9 has a low crystallinity, thereby weakening the mechanical properties for TOCN-9 samples. It is evident that the mechanical properties of TOCN-*x* film exhibited an initial increase followed by a subsequent decrease as the LiCl concentration increased (Figure 5a, Table 3). When dealing with a concentration of 7% LiCl, the tensile strength and Young’s modulus of TOCN-7 reached their maximum values (Table 3), followed by TOCN-5.

The mechanical properties, when combined with the morphological characteristics, enable us to concluded that the TOCN-5 film has better comprehensive mechanical properties in relation to the other samples, which also suggests that the optimal concentration of LiCl in the DMAc/LiCl treatment system for *Camellia oleifera* fruit shell cellulose is 5 wt%. The modification of TOCN-5 with TS was implemented as a consequence. In comparison to the TOCN-5 film, TS-TOCN exhibited a decrease in the tensile strain at break by 39% due to the increased crystallinity along with the deteriorated flexibility (Figure 5b).

### 3.7. Antibacterial Analysis

**Figure 6:**
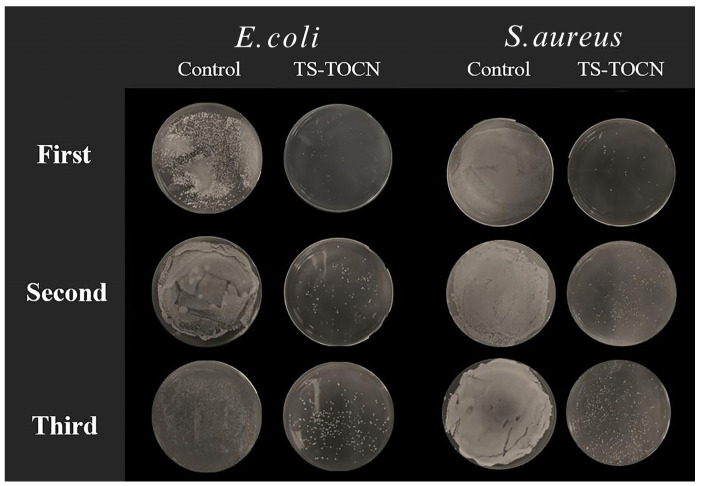
Consecutive antibacterial test results for TS-TOCN films. The bacterial solution was diluted 100 times after co-incubation with the films.

**Figure 7:**
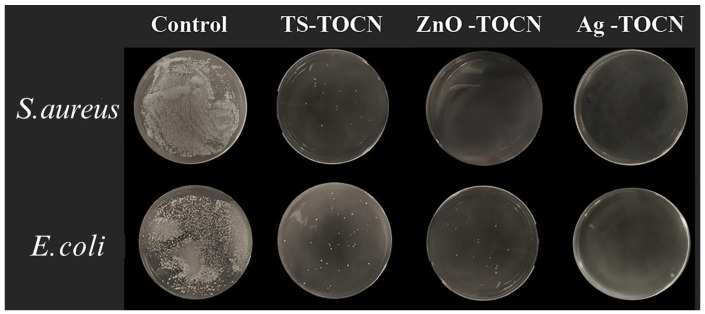
Comparison of antibacterial test results between TS−TOCN film, ZnO−TOCN film, and Ag−TOCN film. The bacterial solution was diluted 100 times after co-incubation with the films.

As depicted in Figure 6 and Figure 7, the TOCN-5 film and TS-TOCN film were tested for their antibacterial efficacy against *E. coli* and *S. aureus*. The results demonstrated that TS-TOCN exhibited potent antimicrobial activity against both *E. coli* and *S. aureus*, particularly toward *S. aureus*, which aligns with previous findings by Zhan Y. et al. [52]. Even after three rounds of antibacterial treatment, TS-TOCN still displayed inhibition effects on bacteria (Figure 6), indicating its exceptional durability as a material-based antibacterial agent.

Furthermore, in comparison with nano ZnO-TOCN and nano Ag-TOCN films, the antibacterial performance of TS-TOCN films was consistent with that of nano ZnO-TOCN films, suggesting that TS could serve as a viable alternative to nano ZnO in terms of antibacterial performance (Figure 7). It was observed that the bactericidal effect of TS-TOCN was achieved through disruption of cellular integrity and reduction in barrier properties. The cytomembrane in bacteria was disrupted by TS, resulting in intracellular substances leakage such as proteins and K^+^, ultimately leading to cell death [53]. Moreover, it is worth noting that TS demonstrated no adverse effects on the growth of human astrocytes in primary culture and poses no harm to the human body, thereby making it suitable for application as a novel antibacterial agent within the fields of food and medicine [54].

### 3.8. Oxygen Barrier Properties

**Figure 8:**
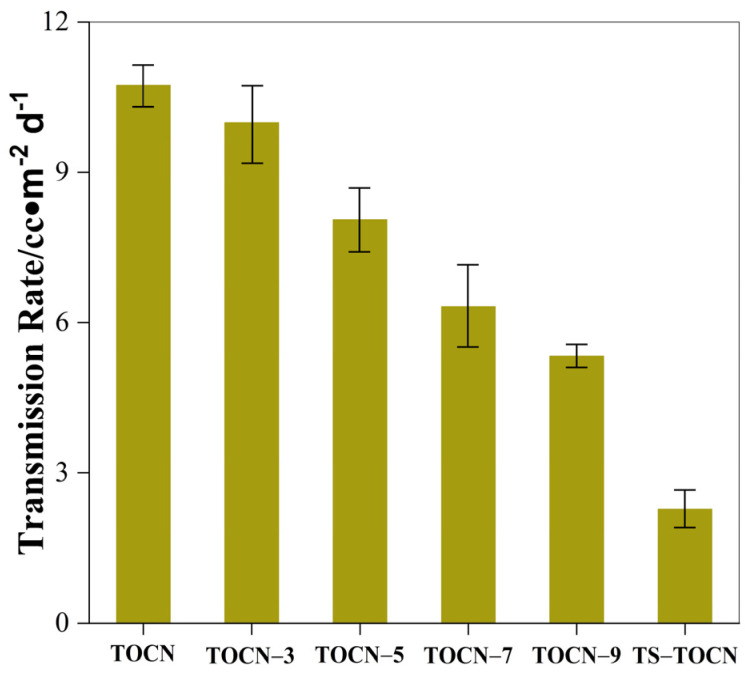
The oxygen permeability of TOCN−*x* films and TS-TOCN film.

The oxygen permeabilities of the TOCN-*x* film and TS-TOCN film are shown in Figure 8. The TOCN-*x* film showed exceptional oxygen barrier properties. Compared with TOCN, the oxygen inhibition properties of films treated with the DMAc/LiCl system were increased by 6%, 24%, 40.9%, and 50.2%. This may be due to the fact that as the concentration of the DMAc/LiCl system increases, the carboxyl group content of the prepared nanocellulose increases, and the average pore size in the system decreases accordingly, making the structure of the membrane denser (Figure 2g–k). The transfer rate of oxygen in the film is reduced, and the high oxygen resistance of the film is realized.

Fukuzumi et al. [55] employed positron annihilation lifetime spectroscopy to measure the average pore size in the TOCN-COONa film and found that it was approximately 0.47 nm, which was slightly larger than the dynamic diameter of oxygen molecules, thus confirming the high resistance to oxygen leakage exhibited by TOCN film. The DMAc/LiCl system treatment enhances the oxygen barrier properties of TOCN films, with an additional improvement observed as the carboxylic acid content increased. Due to their dense structure resulting from ester bonding and hydrogen bonding between the TS molecule and fiber, a tortuous path for the oxygen diffusion is created. Consequently, the TS-TOCN film showed superior oxygen barrier performance compared to TOCN-5, with an oxygen permeability of 2.88 cc·m^−2^·d^−1^ (Figure 8).

### 3.9. Antioxidant Analysis

**Figure 9:**
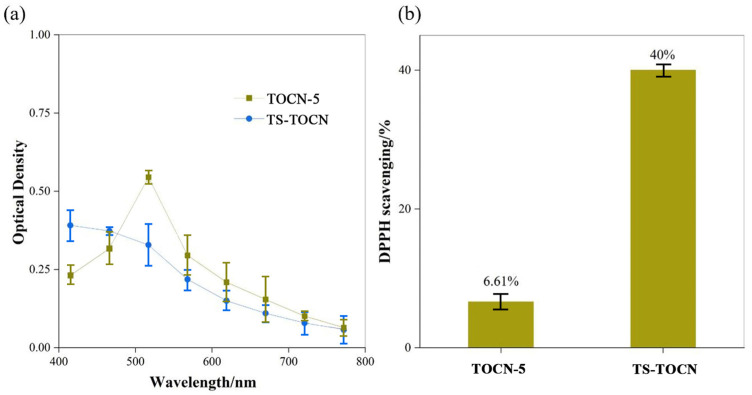
(**a**) UV–vis spectra and (**b**) DPPH scavenging percentage of DPPH solution after being scavenged by TOCN-5 film and TS−TOCN film for 60 min.

The antioxidant effect of TOCN-5 and TS-TOCN was demonstrated after a 60-min treatment, as depicted in Figure 9. DPPH is a stable free radical, and the scavenging ability of antioxidants towards DPPH can be assessed by measuring the degree of light absorption at approximately 517 nm [56]. The pure TOCN-5 samples were regarded as control samples and exhibited no discernible activity in scavenging DPPH radical. However, when grafted with abundant TS, TS-TOCN displayed a remarkable scavenging rate of 40% towards the DPPH solution, indicating its excellent antioxidation effect. This can be attributed to the triterpenoid saponins (TSs) present in TS-TOCN, which induce antioxidant activity by reaction with peroxyl radicals through the hydrogen atom transfer from their hydroxyl groups [57]. Moreover, due to the dose-dependent nature of the antioxidant properties of TS, the scavenging effect on DPPH radicals was further enhanced with an increase in the mass concentration of TS [58]. Consequently, by increasing the grafting rate even more, the antioxidant property of TS-TOCN was improved.

## 4. Conclusions

In summary, we have proposed a rational strategy for the modification of TOCN films with TS to address the waste problem associated with *Camellia oleifera* remainder resources. The utilization of the DMAc/LiCl system effectively destroys the crystalline region of cellulose, resulting in a reduction in crystallinity, thereby increasing the reaction sites for the TEMPO oxidation reaction. The cellulose from the fruit shells of *Camellia oleifera*, pretreated with DMAc/LiCl, was oxidized by TMPO to produce TOCN with a high carboxyl content, thereby facilitating the grafting modification of the TOCN film. Consequently, we successfully developed an antibacterial and antioxidant material based on oil tea remainders. TS-TOCN film demonstrated superior oxygen barrier performance with an oxygen permeability of 2.88 cc·m^−2^·d^−1^. Notably, TS-TOCN film effectively inhibited the growth of both Gram-negative bacteria and Gram-positive bacteria with good repeatability, which is comparable to the antibacterial properties of nano ZnO-TOCN films. In addition, TS-TOCN showed a DPPH scavenging efficiency of 40%, highlighting its excellent antioxidation effect. This work provides a novel and efficient approach for preparing biobased films with bacteriostatic and antioxidant properties, opening up new possibilities for their applications in functional materials.

## Figures and Tables

**Table 1:** Chemical composition of *Camellia oleifera* fruit shells and the treated cellulose. ND: not detectable.

	*Camellia oleifera* Fruit Shell	*Camellia oleifera* Fruit Shell Cellulose	DMAc/LiCl-Treated Cellulose			
			3 wt%	5 wt%	7 wt%	9 wt%
Cellulose %	19.2 ± 0.21	84.3 ± 0.67	87.0 ± 0.32	96.7 ± 0.12	95.0 ± 0.43	96.0 ± 0.52
Hemicellulose %	28.6 ± 0.65	7.3 ± 0.25	5.3 ± 0.25	-	-	-
Holocellulose %	47.9 ± 0.33	-	-	-	-	-
Lignin %	29.1 ± 0.23	3.9 ± 0.30	2.8 ± 0.61	0.7 ± 0.48	-	-
Ash %	3.2 ± 0.31	1.2 ± 0.45	-	-	-	-
Extractive %	19.7 ± 0.69	ND	ND	ND	ND	ND

**Table 2:** The carboxyl content of DMAc/LiCl-treated TOCNs.

Sample	TOCN-0	TOCN-3	TOCN-5	TOCN-7	TOCN-9
Concentration of DMAc/LiCl (wt%)	0	3%	5%	7%	9%
-COOH content (meq/100 g)	160	210	272	326	378

**Table 3:** The mechanical properties of TOCN-x.

	TOCN-0	TOCN-3	TOCN-5	TOCN-7	TOCN-9
Tensile Stress/MPa	34.99967	36.76179	48.15186	63.91672	34.39672
Tensile Strain at Break/%	3.57903	4.18075	4.74976	1.76351	1.80684
Young’s Modulus/MPa	2688	2559	3944	4588	1369

## Data Availability

Data are contained within the article.
